# 55. Impact of Testing Methodology and Reporting on Time to Preferred Antibiotic Therapy in Extended Spectrum Beta-Lactamase producing Enterobacteriaceae (ESBL-E) Bloodstream Infections

**DOI:** 10.1093/ofid/ofab466.257

**Published:** 2021-12-04

**Authors:** Hanh Bui, Frank Tverdek, Stephanie Carnes, Jeannie D Chan, Andrew Bryan, Lori Bourassa, Rupali Jain, Brendon Matsuda-Fong

**Affiliations:** 1 UW Medicine, Seattle, Washington; 2 Fred Hutchinson Cancer Research Center, Seattle, Washington; 3 UW Medicine, Harborview Medical Center, Seattle, WA; 4 University of Washington, Seattle, Washington; 5 University of Washington School of Medicine, Seattle, WA

## Abstract

**Background:**

Harborview Medical Center (HMC) identifies organisms and an ESBL genotype (CTX-M) via Verigene® Gram-Negative Blood Culture Nucleic Acid Test (BC-GN). University of Washington-Montlake (UWML) uses matrix-assisted laser desorption ionization time-of-flight mass spectrometry (MALDI-TOF MS) for organism identification directly from positive blood cultures and ceftriaxone results by Kirby Bauer disk diffusion (KB) are reported 18 hours later. No ESBL comment is reported at UWML. We aimed to determine whether the methodology in identification and reporting of ESBL-E from blood cultures between two hospitals has an impact on time to preferred therapy with a carbapenem antibiotic.

**Methods:**

Retrospective observational study conducted at UWML and HMC in Seattle, WA between 1/10/2015 and 9/15/2020. Adult patients were eligible if they had ≥1 positive blood culture with an Enterobacteriaceae isolate resistant to ceftriaxone and were on antibiotic treatment. The primary outcome was the difference in time to preferred definitive therapy with a carbapenem antibiotic in patients an ESBL-E bloodstream infection (BSI) identified by Verigene® vs. MALDI-TOF MS/KB.

**Results:**

A total of 199 patients were screened; 67 were included for UWML and 68 at HMC. The average time to initiation of a carbapenem antibiotic was 42 ±26.5 hours at UWML and 28 ±19.7 hours at HMC. A t-test detected a difference in time to preferred therapy between a Verigene® vs. MALDI-TOF MS/KB tested ESBL-E BSI [95% confidence interval (CI), 5.3-22.9]. The hazard ratio to carbapenem initiation for HMC is 1.73643 [95% CI, 1.1405-2.644].

**Conclusion:**

A statistically significant difference in time to preferred definitive therapy among patients with an ESBL-E BSI processed by Verigene® was found compared to MALDI-TOF MS. The results suggest standardization in protocols between the UWML and HMC hospitals is warranted.

**Disclosures:**

**Andrew Bryan, MD, PhD**, **Shionogi Inc.** (Grant/Research Support)

